# Correction to ‘BMAL1 Deficiency Promotes Skeletal Mandibular Hypoplasia via OPG Downregulation’

**DOI:** 10.1111/cpr.70163

**Published:** 2026-01-26

X. Zhou, R. Yu, Y. Long, et al., “BMAL1 Deficiency Promotes Skeletal Mandibular Hypoplasia via OPG Downregulation,” *Cell Proliferation* 51 (2018): e12470, https://doi.org/10.1111/cpr.12470.

(1) The images in Figure 5A of RAW+BMSCs OPG (0 ng/mL) and RAW+(MC3T3‐shRNA#1) OPG (50 ng/mL) appeared to overlap.

(2) The TRAP Staining image appeared incorrectly in Figure 6C (Wild Type twice a week, 6 weeks, OPG 0 ng/mL).

Corrected Figures 5A and 6C are provided below. The correction does not alter any findings and conclusions reported in this article.

**Figure 5A:**
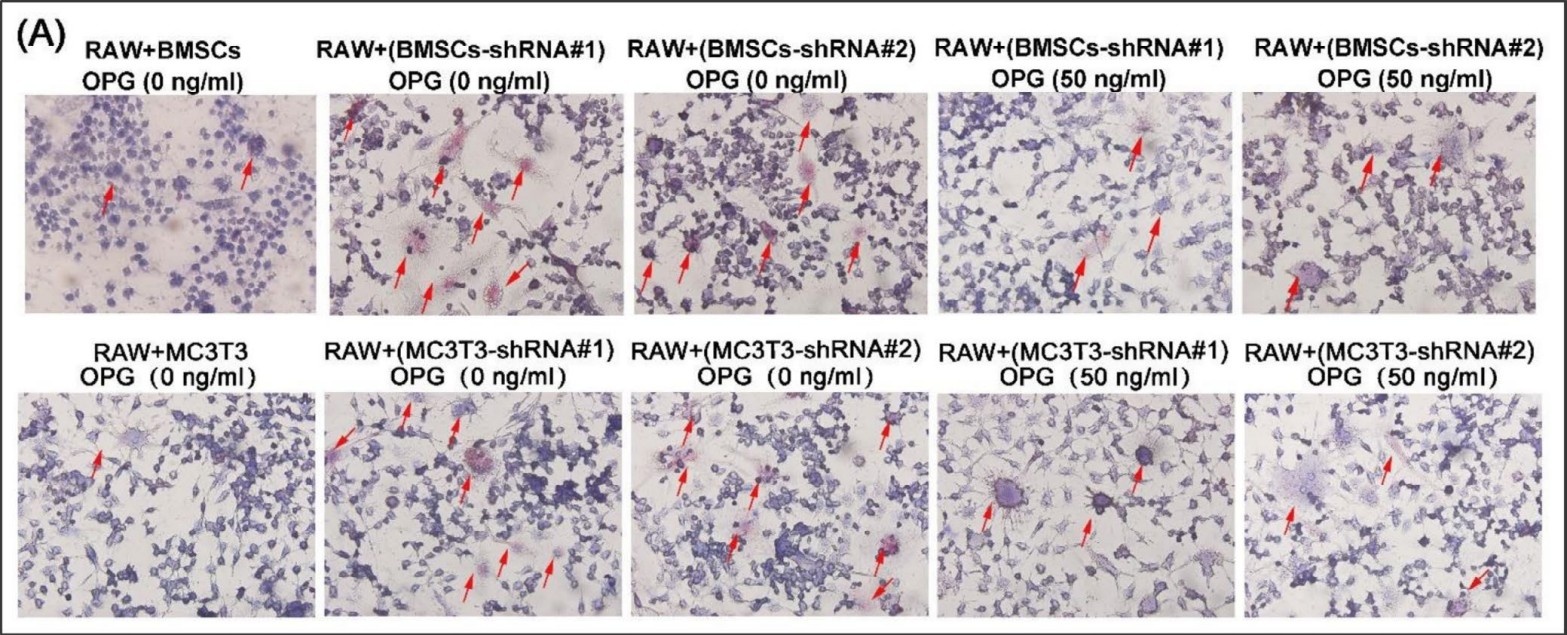


**Figure 6C:**
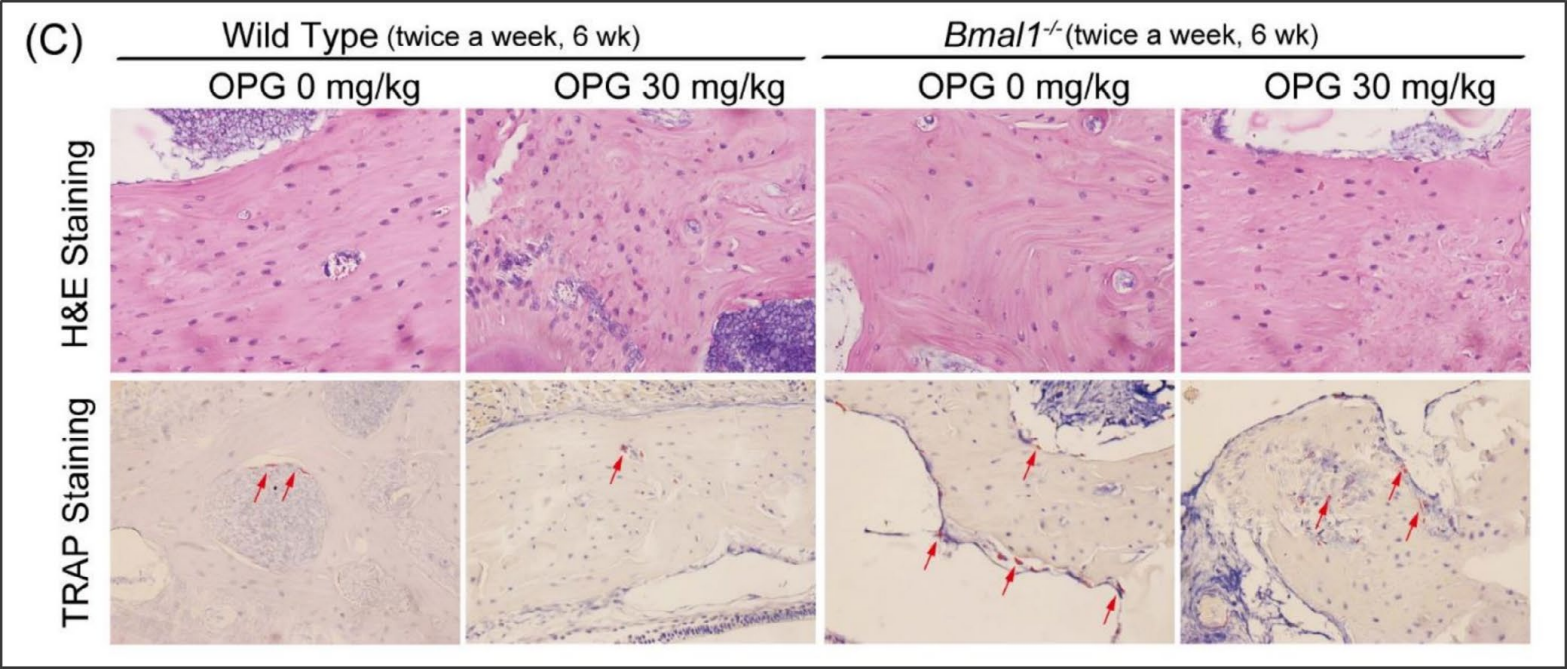


We apologise for these errors.

